# Associations between prenatal paracetamol exposure and brain development from ages 4–16: A longitudinal MRI study

**DOI:** 10.1016/j.dcn.2025.101667

**Published:** 2025-12-30

**Authors:** Stine Kleppe Krogsrud, Hedvig Nordeng, James M. Roe, Janne von Koss Torkildsen, Mollie E. Wood, Eivind Ystrom

**Affiliations:** a Center for Lifespan Changes in Brain and Cognition (LCBC), Department of Psychology, University of Oslo, Norway; b School of Health Sciences, Kristiania University of Applied Sciences, Oslo, Norway; c Pharmacoepidemiology and Drug Safety Research Group, Department of Pharmacy, University of Oslo, Norway; d Department of Child Health and Development, Norwegian Institute of Public Health, Oslo, Norway; e PROMENTA Research Center, Department of Psychology, University of Oslo, Norway; f Department of Special Needs Education, University of Oslo, Norway; g Department of Epidemiology, Gillings School of Global Public Health, University of North Carolina at Chapel Hill, United States; h Computational Radiology and Artificial Intelligence, Department of Radiology and Nuclear Medicine, Oslo University Hospital, Norway; i Centre for Research on Equality in Education (CREATE), Faculty of Educational Sciences, University of Oslo, Norway

**Keywords:** Brain morphology, In utero exposure, Neurodevelopment, Paracetamol, Working memory

## Abstract

Research has raised concerns regarding the potential impact of prenatal paracetamol exposure on fetal neurodevelopment. This is the first longitudinal study to investigate whether such exposure is related to structural brain development and cognitive abilities during childhood and adolescence. The sample includes 447 children aged 4.1–16.2 years with 905 MRI scans. The study is based on The Norwegian Mother, Father and Child Study (MoBa), and uses data from the Medical Birth Registry of Norway. The exposure variable is mothers’ self-reported use of paracetamol from prenatal and postnatal questionnaires. Among 447 children (229 girls; mean age 8.38 years), 193 (43.2 %) were prenatally exposed. Days of exposure range from 1 to 128 (mean 8.2). Vertex-wise linear mixed effect models showed that exposed children (n = 193; 393 scans) had slightly smaller cortical surface area and volume in some regions compared to non-exposed children. These results were seemingly driven by long-term exposed children (≥14 days *in utero*; n = 35; 72 scans, and exposed in all three trimesters, n = 21; 44 scans). However, group differences in brain structure were small with largely overlapping distributions, and the observational design and risk of unmeasured confounding preclude causal inference. Thus, the clinical significance remains uncertain. For cognitive abilities, results were reassuring showing no significant effect of prenatal paracetamol exposure on working memory capacity or IQ scores.

## Highlights

• We examined the effect of prenatal paracetamol exposure on brain and cognition.
• This is a longitudinal study including children from 4 to 12 years of age.
• Children exposed *in utero* had slightly smaller cortical surface area and volume.
• Differences in brain structure were more apparent with long-term prenatal exposure.
• No association was found between prenatal exposure and IQ or working memory capacity.

## 1 Introduction

Paracetamol is one of the most commonly used medications in pregnancy, used by 40 %-65 % of pregnant women (Liew and Ernst, 2021, Lupattelli et al., 2014, Werler et al., 2005), and currently recommended as a first-line drug for pain and fever treatment during pregnancy. However, concerns over the long-term effects of prenatal exposure on neurodevelopmental outcomes have risen during the past decades (Avella-Garcia et al., 2016, Bauer and Kriebel, 2018, Brandlistuen et al., 2013, Liew et al., 2016a, Liew et al., 2014, Masarwa et al., 2018, Nilsen et al., 2023, Olsen and Liew, 2017, Stergiakouli et al., 2016, Thompson et al., 2014, Vlenterie et al., 2016, Ystrom et al., 2017). In 2021, Bauer et al. (2021), called for precautionary action by increasing awareness among health professionals and pregnant women about the potential adverse effects of paracetamol on offspring. Others claim that the evidence supporting an increased risk of adverse neurodevelopmental outcomes during childhood following *in utero* exposure to paracetamol is weak, inconsistent, and with methodological limitations (ENTIS, 2021; Gilman and Hornig, 2019).

The association between prenatal paracetamol exposure and adverse neurodevelopmental outcomes has been investigated in several observational studies including cohorts with over 220,000 mother–child pairs (Bauer and Kriebel, 2018, Hjorth et al., 2019, Liew and Ernst, 2021). Of these, several cohort studies have found that paracetamol use during pregnancy may be associated with a slightly higher risk of hyperkinetic disorders and attention-deficit hyperactivity disorder (ADHD) (Gustavson et al., 2021, Ji et al., 2020, Ji et al., 2018, Liew et al., 2014, Thompson et al., 2014, Trønnes et al., 2020, Ystrom et al., 2017). In a recent magnetic resonance imaging (MRI) study, Baker et al. (2020) found that prenatal paracetamol exposure was associated with increased risk of ADHD and increased negative resting state connectivity between the left prefrontal cortex and the right precentral gyrus at ages 6 and 7 years. In addition, a sibling-controlled cohort study from the Norwegian Mother, Father and Child Cohort Study (MoBa), showed that use of paracetamol for ≥ 28 days during pregnancy was associated with adverse developmental outcomes such as poorer gross motor development, communication skills and externalizing problems at 3 years of age compared to siblings non-exposed or exposed to fewer days of paracetamol (Brandlistuen et al., 2013). Further, prenatal paracetamol exposure has been associated with lower intelligence quotient (IQ) (Liew et al., 2016b) and poorer attention (Avella-Garcia et al., 2016), executive functions (Liew et al., 2016a, Liew et al., 2016b, Rifas-Shiman et al., 2020), and language delay during childhood (Bornehag et al., 2018). However, there have also been several studies showing no association between prenatal paracetamol exposure and adverse neurodevelopmental outcomes in children (Bertoldi et al., 2020, Laue et al., 2019, Tovo-Rodrigues et al., 2020). What is more, Ystrom et al. (2017) found *paternal* long term use of paracetamol before pregnancy to be equally associated with offspring ADHD diagnosis as maternal long term use during pregnancy, suggesting genetic confounding rather than any causal effect.

Crucially, it is unknown whether prenatal exposure to paracetamol may affect fetal brain development with potential long-term effects because no longitudinal MRI study has investigated the effect of paracetamol on structural brain development. Most recently, Walhovd et al. (2024) demonstrated that prenatal growth, as indexed by birth weight, showed stable, lifelong associations with cortical surface area and volume across the lifespan, and the effects did not consistently influence rates of brain change. Early-life conditions, such as birth weight and parental education, have also been linked to stable brain–cognition relationships lasting into old age (Walhovd et al., 2016). Together, these studies highlight the lifelong impact of prenatal factors on brain structure and function. Moreover, studies in mice indicate that prenatal paracetamol exposure can similarly have lasting neurodevelopmental effects (Viberg et al., 2014). Accordingly, our study aimed to examine whether maternal self-reported prenatal paracetamol exposure is associated with cortical development and cognitive function in children aged 4–16 years. IQ and working memory (WM) are of particular interest, as several studies have reported associations between prenatal paracetamol exposure and cognitive outcomes in childhood, such as lower IQ, and impairments in attention, executive functions and language acquisition (Avella-Garcia et al., 2016, Liew et al., 2016a, Liew et al., 2016b, Rifas-Shiman et al., 2020, Bornehag et al., 2018). WM is a core component of executive function that supports reasoning, attention regulation, and learning, and shows strong associations with IQ during development (Best and Miller, 2010, Fry and Hale, 2000, Gathercole et al., 2004). Deficits in WM have consistently been observed in children with attentional and executive difficulties (Gathercole and Alloway, 2006, Martinussen et al., 2005). Including both brain and cognitive measures therefore allows for a more comprehensive assessment of whether prenatal paracetamol exposure is associated with alterations in neurodevelopment.

Structural brain development follows nonlinear age trajectories. During early childhood, surface area, volume and cortical thickness all increase and reach their peaks at different ages: cortical thickness peaks around 2 years of age, total cortical volume around 6 years, and cortical surface area around 11–12 years (Bethlehem et al., 2022). Following these peaks, all measures show nonlinear decreases during childhood and throughout life (Krogsrud et al., 2021, Mills et al., 2016, Tamnes et al., 2017, Walhovd et al., 2016). As there are no structural MRI studies investigating the effect of prenatal paracetamol exposure on brain development (Baker et al., 2020), our hypotheses were based on prior research on structural brain development and the long-lasting impact of early-life factors on brain structure (Bethlehem et al., 2022, Krogsrud et al., 2021, Mills et al., 2016, Tamnes et al., 2017, Walhovd et al., 2012, Walhovd et al., 2016), and structural findings in ADHD (Carmona et al., 2005, Ellison-Wright et al., 2008, Hoogman et al., 2017, Hoogman et al., 2019). We hypothesized that 1) prenatal paracetamol exposure will be associated with smaller cortical surface area and volume, and thicker cortex in the developing brain, and 2) this association will depend on the duration of exposure during fetal life (Brandlistuen et al., 2013, Gervin et al., 2017, Gustavson et al., 2021, Liew et al., 2016a, Vlenterie et al., 2016). Further, we expected 3) paracetamol exposure during fetal brain development to have a continuous impact on brain development in childhood, yielding different offsets (i.e., different starting points), yet similar developmental slopes (i.e., the same rate of change with age) (Viberg et al., 2014, Walhovd et al., 2016, Walhovd et al., 2024), 4) and that long term exposure would be associated with lower IQ and poorer working memory (WM) capacity compared to non-exposed children (Liew et al., 2016a).

## 2 Materials and methods

### 2.1 Participants

The study is based on a subsample from The Norwegian Mother, Father and Child Cohort Study (MoBa) (Magnus et al., 2016, Magnus et al., 2006), and uses data from the Medical Birth Registry of Norway (MBRN). MBRN is a national health registry containing information about all births in Norway. MoBa is a population-based pregnancy cohort study conducted by the Norwegian Institute of Public Health. Participants were recruited from all over Norway from 1999 to 2008. The women consented to participation in 41 % of the pregnancies. The cohort includes approximately 114,500 children, 95,200 mothers and 75,200 fathers. The current study is based on version 8 of the quality-assured data files released for research in 2015. The establishment of MoBa and initial data collection was based on a license from the Norwegian Data Protection Agency and approval from The Regional Committees for Medical and Health Research Ethics. The MoBa cohort is currently regulated by the Norwegian Health Registry Act. The current study was approved by The Regional Committees for Medical and Health Research Ethics (10491). A subsample of children (2000 children from the Oslo area, and 700 children from the Trondheim area) were invited to participate in the MoBa Neurocognitive development (MoBaNeuroCog) project at the Center for Lifespan Changes in Brain and Cognition (LCBC) (Krogsrud et al., 2016). Of these, 523 children aged 4.1–10.6 years participated and were scanned using MRI and tested on several neuropsychological tests. The children were followed longitudinally at three timepoints in Oslo, and two timepoints in Trondheim. The full longitudinal sample ranges from 4.1 to 16.2 years of age and has 1123 MRI scans. Participants’ age at the three timepoints is shown in Table 1. Written informed consent was obtained from the parent/guardian for all participants, and from participants ≥ 12 years of age. Oral assent was given by participants < 12 years at all time points. A parent of each participant completed a structured interview to ascertain participant eligibility at all time points. Included participants were required to be fluent Norwegian speakers and have normal or corrected-to normal vision and normal hearing.

**Table 1:** Full sample characteristics for each timepoint of assessment.

Time points	N	aAge	IQ	Working memory
	(female)	Mean (SD, range)	Mean (SD, range)	Mean (SD, range)
1	410 (206)	6.7 (1.4, 4.1–10.6)	109 (13.2, 73–143)	10.1 (3.1, 0–21)
2	305 (165)	8.3 (1.4, 5.5–12.1)	110 (12.5, 74–142)	11.3 (2.8, 6–19)
3	178 (98)	12.4 (1.3, 10.5–16.2)	109 (11.7, 77–141)	14.7 (3.1, 9–25)

a Age refers to age (years) at cognitive testing.

### 2.2 Exclusion criteria

**Fig. 1:**
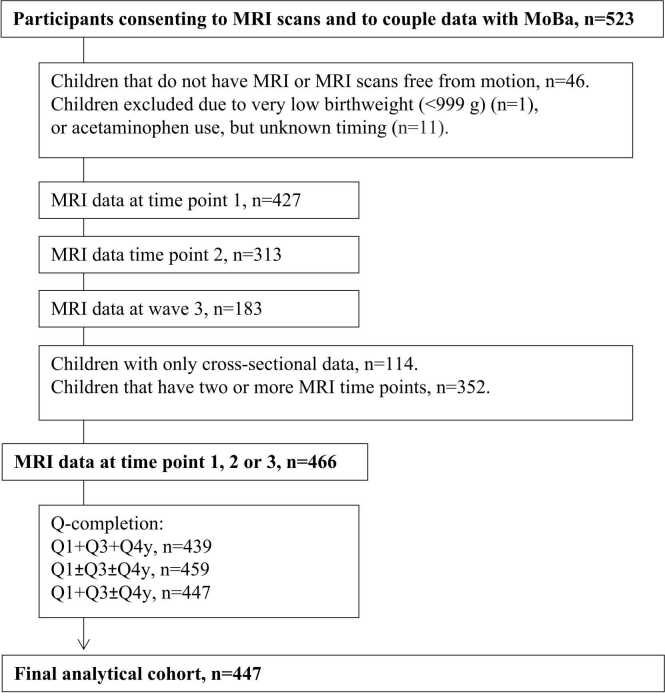
**Flow-chart.** Flow-chart to achieve final analysis cohort.

Participating children were excluded from the current study if they had not completed at least one structural MRI scan, and if no structural MRI scans were free from MRI contraindications (n = 46), if the timing of paracetamol exposure was completely unknown (n = 11), if they had very low birthweight (<999 g) and/or were born before 28 weeks gestation (n = 1). MRI scans were evaluated by a neuroradiologist and required to be free of significant injuries or conditions. In addition, children with no information about prenatal medication exposure during week 18 and week 30 of gestation were excluded. After applying these criteria, the number of unique children was reduced to 447 (229 females [51.2 %]), with in total 905 MRI scans. An overview of the sample including number of MRI scans and MoBa questionnaires is displayed in Fig. 1.

### 2.3 Paracetamol exposure measures

Information on maternal self-reported paracetamol (ATC code NO2BE01) use was obtained through MoBa questionnaires. Paracetamol use was available from two prenatal and one postnatal questionnaire. At week 18, week 30, and 6 months postpartum, the mothers reported details on medication use specifically for each medical condition. In addition, at each time point, the mothers listed names of any additional medications used. For each indication, the mother could name the medication taken in an open textbox and specify the following exposure windows: 6 months before gestation; gestational weeks 0–4, 5–8, 9–12, and ≥ 13 (until completion of the first questionnaire); 13–16, 17–20, 21–24, 25–28, and ≥ 29 (until completion of the second questionnaire); and ≥ 30 (until birth), 0–3 months postpartum, and 4–6 months postpartum. Questionnaires can be found at https://www.fhi.no/en/studies/moba/for-forskere-artikler/questionnaires-from-moba/. Number of days of exposure was reported as a total across all exposure windows in each questionnaire. In the current study, the exposure variable includes paracetamol exposure during first, second and/or third trimester, and a few cases where timing of exposure during pregnancy was unknown (n = 3). Number of days of exposure ranged from 1 to 128 (mean 8.21). The timing and duration of paracetamol exposure during pregnancy is shown in Table 2. In all statistical analyses, we treated prenatal paracetamol exposure as binary and compared to non-exposed participants (controls). We did this across 3 varying levels of exposure (note therefore that some participants overlap across exposure groups); 1) Exposed at any time during pregnancy (n = 193; 393 MRI scans), 2) exposed for ≥ 14 days (n = 35; 72 MRI scans), or 3) exposed in all three trimesters (n = 21; 43 MRI scans). In all analyses, these three exposure groups were compared to the group of children not exposed to paracetamol during pregnancy (n = 245; 512 MRI scans).

**Table 2:** Various patterns of paracetamol exposure during pregnancy in the analytic study sample (n = 447).

**Exposure pattern**		**N total= 193, n (%)**
BY DURATION	Number of trimesters;	
	Use in minimum one trimester	193 (100.0)
	Use in minimum two trimesters	93 (48.2)
	Use in all three trimesters	21 (10.9)
	aDays;	
	1–7 days	130 (67.4)
	≥ 7 days	63 (32.6)
	< 14 days	152 (78.8)
	≥ 14 days	35 (18.1)
	< 21 days	171 (88.6)
	≥ 21 days	16 (8.3)
BY TIMING	Trimester;	
	Use in only 1st trimester	38 (19.7)
	Use in only 2nd trimester	49 (25.4)
	Use in only 3rd trimester	13 (6.7)
	bGestational week;	
	GW 0–4	23
	GW 5–8	51
	GW 9–12	89
	GW 13–16	95
	GW 17–20	33
	GW 21–24	40
	GW 25–28	45
	GW 29–34	20
	GW 35-delivery	36

a The total number of days of paracetamol exposure in the sample was 128 (mean 8.2).

b Percent is not reported for gestational weeks as all values are< 2 %.

### 2.4 Cognitive abilities

IQ measures: For children above 6.5 years of age, full-scale IQ was calculated from the Wechsler Abbreviated Scale of Intelligence (WASI), which comprises the four subtests Similarities, Vocabulary, Block Design and Matrix Reasoning (Wechsler, 1999). For children 6.5 years of age or younger, full-scale IQ was calculated as the mean of Verbal and Performance IQ from the four subtests Similarities, Vocabulary, Block Design and Matrix Reasoning of Wechsler Preschool and Primary Scale of Intelligence (WPPSI) (Wechsler, 2002). All full-scale IQ measures are standardized IQ index scores that are age-adjusted. Full-scale IQ scores at all three timepoints are shown in Table 1. IQ scores for the various exposure groups are shown in Table 3.

**Table 3:** Sample characteristics of the analytic study population.

	**Non-exposed** **N = 254**	**Paracetamol exposed, full sample** **N = 193**	**Paracetamol exposed for ≥ 14 days** **N = 35**	**Paracetamol exposed in all 3 trimesters** **N = 21**
	**No. (%)** **Mean, SD (Range)**	**No. (%)** **Mean, SD (Range)**	**No. (%)** **Mean, SD (Range)**	**No. (%)** **Mean, SD (Range)**
Sex (female)	125 (49.2)	104 (53.9)	18 (51.4)	7 (33.3)
aCo-medications used during pregnancy	15 (5.9)	15 (7.8)	3 (8.6)	4 (19.0)
Paracetamol use prior to pregnancy	42 (16.5)	77 (39.9)	21 (60.0)	11 (52.4)
Married/cohabiting	241 (94.9)	188 (97.4)	34 (97.1)	21 (100)
Smoking during pregnancy	13 (5.2)	12 (6.2)	3 (8.8)	1 (4.8)
Alcohol intake during pregnancy	51 (21.2)	38 (21.2)	6 (20.7)	4 (21.5)
Fever during pregnancy	23 (9.1)	16 (8.3)	5 (14.1)	2 (9.5)
Pain during pregnancy	182 (71.7)	168 (87.0)	34 (97.1)	21 (100.0)
Days of exposure	NA	8.2, 10.7 (1–128)	28.8, 13.1 (14–128)	23.1, 10.8 (3–128)
Maternal age at delivery (years)	31.8, 4.5 (20–43)	31.3, 4.4 (17–42)	30.7, 4.7 (20–41)	30.2, 4.2 (22–39)
bMaternal education	5.2, 0.90 (1–6)	5.2, 0.88 (1–6)	5.0, 1.0 (1–6)	5.2, 0.9 (1–6)
Birth weight (gram)	3588, 519 (1564–4950)	3673, 575 (1620–5480)	3774, 584 (2827–5010)	3667, 619 (2430–5010)
Gestational age (days)	280, 11.4 (228–298)	280, 11.6 (213–298)	281, 10 (248–298)	281, 10.7 (255–297)
Child age (years)	8.38, 2.4 (4.2–16.2)	8.3, 1.57 (4.2–16.1)	8.2, 2.5 (4.2–16.1)	8.2, 2.6 (4.6–16.1)
cFull scale IQ	109, 13.4 (73–143)	109, 12.7 (77–143)	107, 12.8 (79–125)	106, 9.3 (88–123)
Digit Span score	10.2, 3.1 (0–18)	10.2, 3.1 (3–21)	10.2, 3.5 (4–21)	10.2, 3.2 (4–17)

Missingness: maternal education level was missing for n = 5, information about maternal alcohol intake was missing for n = 27, and information about maternal smoking was missing for n = 3.

a Co-medication includes: NSAIDs (M01A, N02BA), opioids (N02A), triptans (N02CC) and antiepileptics (N03A).

b Maternal education is classified from 1 to 6; 1) 9-year primary and lower secondary school, 2) 1–2 year upper secondary school, 3) Vocational high school, 4) 3-year junior college, 5) University college or university, 4-year degree, 6) University college or university, more than 4-year degree.

c Full scale IQ scores from all three time points.

Working memory performance: Verbal WM was assessed with diﬀerent versions of the Digit Span subtest, all with the same number of sequences and sequence lengths. The versions used were the Wechsler Intelligence Scale for Children Third Edition (WISC-III) (Wechsler, 1991), Wechsler Adult Intelligence Scale - Third and Fourth Edition (WAIS-III/WAIS-IV) (Wechsler, 1987), and Wechsler Memory Scale–Revised (WMS–R) (Wechsler, 1987). The children were verbally presented with numeric sequences of increasing length. In the ﬁrst part of the test, they were required to repeat the digits in the same order as presented (Digit Span Forwards). In the second part, they were asked to repeat the digits in reversed presentation order (Digit Span Backwards). The length of the digit sequences increased every other sequence. The stop criterion was two wrong answers within a pair of equal length. Digit Span Total, the variable used in the current study, was calculated from Digit Span Forwards and Digit Span Backwards scores. WM scores at all three timepoints are shown in Table 1. WM scores, based on all timepoints, for the various exposure groups are shown in Table 3. WM scores were missing for 7 children (1.6 %), exposed: n = 2 (1.0 %); non-exposed: n = 5 (2.0 %), and IQ scores were missing for 10 children (2.2 %), exposed: n *=* 3 (1.6 %); non-exposed: n = 7 (2.8 %).

### 2.5 Covariates

Covariates were chosen based on established associations with both maternal medication use and child neurodevelopmental outcomes (Hornig et al., 2018, Moylan et al., 2015, Nygaard et al., 2018, Vejrup et al., 2022, Walhovd et al., 2016, Walhovd et al., 2024). These included prenatal co-medication, alcohol consumption and smoking during pregnancy, maternal education, maternal age at delivery, fever/pain conditions, paracetamol use 6 months prior to pregnancy, birth weight, child sex and scanner site. An overview of all covariates for the full sample, for children exposed in≥ 14 days, and for children exposed in all three trimesters are shown in Table 3. Below, we provide a clarification of how the covariates were categorized:

Co-medication*:* The following co-medications were investigated in the current sample: non-steroidal anti-inflammatory drugs (NSAIDs) (ATC code M01A (e.g. diclofenac, ibuprofen, and naproxen) and N02BA (aspirin), opioids (N02A), triptans (N02CC), antiepileptics (N03A), antipsychotics (N05A), antidepressants (N06A), benzodiazepines (N05CD and N05BA), benzodiazepine-like drugs (N05CF), and stimulants (N06BA). To preserve confidentiality, medications used by< 10 participants (NSAIDs, opioids, triptans, antiepileptics) are reported all together as co-mediation.

Education level*:* Maternal education level was reported at week 18 during pregnancy. Education level was coded as the highest level of completed or ongoing education. 1) 9-year primary and lower secondary school, 2) 1–2 year upper secondary school, 3) Vocational high school, 4) 3-year junior college, 5) University college or university, 4-year degree (Bachelor’s degree, nurse, teacher, engineer), 6) University college or university, more than 4-year degree (Master’s degree, medical doctor, PhD).

Alcohol use during pregnancy: Maternal alcohol intake was classified as “yes” or “no“, independent of which trimester, amount and frequency.

Smoking during pregnancy*:* Maternal smoking was classified as “yes” or “no” independent of which trimester, amount and frequency.

Fever and pain during pregnancy*:* Maternal fever and experiencing pain was classified as “yes” or “no” independent of paracetamol use during pregnancy. Migraine was included in the pain condition.

Patterns of missingness were as follows; maternal education level was missing for n = 5 (1.1 %), information about maternal alcohol intake was missing for n = 27 (6.0 %), and information about maternal smoking was missing for n = 3 (0.7 %). For these covariates, we created a separate “missing” category by coding missing values as their own dummy variable. These missingness indicators were included in all statistical models, allowing participants with incomplete covariate information to be retained in the analyses.

### 2.6 MRI acquisition and preprocessing

Imaging data were acquired from three different Siemens scanner models in Norway: Avanto 1.5 Tesla (T) at St. Olavs Hospital in Trondheim, and Avanto 1.5 T, Skyra 3 T and Prisma 3 T at Oslo University Hospital Rikshospitalet. A total of 715 scans were collected on Avanto scanners, of which 229 were collected in Trondheim. On the two Avanto scanners, identical T1 weighted MPRAGE’s were collected with the following parameters: TR: 2400 ms, TE: 3.61 ms, TI: 1000 ms, flip angle: 8°, slice thickness: 1.2 mm, FoV: 240 × 240, and 160 slices. There were 12 scans collected at the Skyra with the following parameters: TR: 2300 ms, TE: 2.98 ms, TI: 850 ms, flip angle: 8°, slice thickness: 1 mm, FoV: 256 × 256, and 176 slices. On the Prisma, there were 178 scans with the following parameters: TR: 2400 ms, TE: 2.22 ms, TI: 1000 ms, flip angle: 8°, slice thickness: 0.8 mm, FoV: 240 × 256, and 208 slices. For the youngest children, integrated parallel acquisition techniques (iPAT) were used, acquiring multiple T1 scans within a short scan time, enabling us to discard scans with residual movement. Previous studies have shown that accelerated imaging does not introduce measurement bias in surface-based measures when using FreeSurfer for image analysis, compared with a standard MPRAGE protocol with otherwise identical voxel dimensions and sequence parameters (Wonderlick et al., 2009). All scans were visually rated for movement, and only scans with rating 1–2 on a 4-point scale were included (no visible or only very minor possible signs of movement), as movement is a major concern (Reuter et al., 2015). When multiple acquisitions were available, the single best-quality scan was selected. While this conservative approach minimizes overt motion artifacts, we note that it does not capture micro-movements that may remain undetectable on visual inspection.

MRI data were processed and analyzed with FreeSurfer 6.0 (Dale et al., 1999, Fischl et al., 1999, Fischl et al., 2004) (http://surfer.nmr.mgh.harvard.edu/), using the longitudinal stream on all participants (Reuter et al., 2012). This procedure yields a measure of cortical thickness for each person at each point on the reconstructed surface and is capable of detecting sub-millimeter differences between groups (Fischl and Dale, 2000, Kuperberg et al., 2003, Rosas et al., 2002). The processing steps include removal of non-brain tissue (Segonne et al., 2004), automated Talairach transformation, and intensity correction (Sled et al., 1998). Intensity and continuity information from the 3D volume are used in segmentation and deformation procedures to reconstruct a gray/white and gray/cerebrospinal fluid boundary throughout the brain (Dale et al., 1999, Fischl et al., 2002, Fischl et al., 2004). Cortical surfaces then undergo inflation, registration to a spherical atlas, and identification of gyral and sulcal regions (Desikan et al., 2006, Fischl et al., 2004). Specific advantages of the longitudinal stream include that an unbiased within-subject template space and image (Reuter et al., 2012) is created using robust, inverse consistent registration (Reuter et al., 2010). Several of the usual processing steps, such as skull stripping, Talairach transforms, atlas registration as well as spherical surface maps and parcellations are then initialized with common information from the within-subject template, significantly increasing reliability and statistical power (Reuter et al., 2012). Smoothing using a circularly symmetric Gaussian kernel with a full width at half maximum (FWHM) of 15 mm was used for volume, surface area and cortical thickness maps.

Different scanners have been found to yield different thickness estimates but not to bias the correlation with external measures such as cognitive test scores or skew the rank-order between participants (Dickerson et al., 2008). Thus, we expect that including scanner as a covariate of no interest in the analyses will remove most of the effects on brain structure estimates resulting from the use of different scanners. This assumption has been tested in the current sample showing no significant differences in estimated mean cortical thickness between scanners (Krogsrud et al., 2021).

### 2.7 Statistical analyses

#### 2.7.1 Age trajectories

MoBa data was processed using IBM SPSS Statistics 24.0 (IBMCorp, 2020) and Stata (StataCorp, 2019) to generate paracetamol exposure variables. Generalized additive mixed models (GAMMs) were run with R v4.0.0 (R R Core Team, 2020) using the «gamm4 » package (Wood and Scheipl, 2017). The «itsadug» package was used to facilitate interpretation of model results, and visualization was performed with the «ggplot2 » package (v3.3). First, GAMMs were run to estimate age-trajectories for total cortical area, volume, mean cortical apparent thickness, as well as for WM capacity, in the full MoBaNeuroCog subsample. Subsequently, GAMMs were used to estimate age trajectories in these variables for the exposed (n = 193) and non-exposed group (n = 254), test group-differences in the main effect of exposure, and group-differences in the non-linear slope of age (age x exposure), between non-exposed and exposed children. These were repeated for the children exposed for ≥ 14 days (n = 35) and children exposed in all three trimesters (n = 21). GAMMs are well-suited to characterize trajectories of brain variables which are often non-linear (Fjell et al., 2010, Sørensen et al., 2020). Participant intercept was included as a random eﬀect, and thin plate regression splines were used for the smooth term of age. The k-parameter was set to the default value, and adequacy of this setting was assessed using the k.check function from the “mgcv” package. Sex, birth weight, prenatal exposure to co-medication, alcohol consumption and smoking during pregnancy, maternal education, maternal age at delivery, fever and pain during pregnancy, paracetamol use 6 months prior to pregnancy and scanner were included as covariates.

Differences in IQ scores were tested with LMEs comparing the non-exposed children and (1) all the paracetamol-exposed children, (2) children exposed for ≥ 14 days, and (3) the children exposed in all three trimesters. Because IQ scores are standardized and typically stable over time, LMEs were deemed appropriate, as nonlinear age effects on these measures were not expected. All covariates were included as before except for the child’s age, because IQ measures are standardized index scores that are already age-adjusted (hence these are visualized using box plots). We applied false discovery rate (FDR)-correction across all analyses of total brain measures and WM (GAMMs) and IQ (LMEs), across 15 tests per effect (main effect and age x exposure; Table 4). Significance was considered at p(FDR)< .05.

**Table 4:** The effect of paracetamol exposure *in utero* vs non-exposure on brain characteristics and cognitive function.

	**The effect of paracetamol exposure*****in utero across age***						**Smooth age x exposure**			
Exposure groups	Brain and cognition	Std.Beta	Std.Error	P-value	95 % CI	aEdf		Ref.df	F	P-value
Total exposure (n = 193)	Area	−0.073	0.084	0.384	−0.239 – 0.092	1.00		1.00	0.002	0.964
	Volume	−0.043	0.085	0.612	−0.210 – 0.123	1.00		1.00	0.235	0.628
	Thickness	0.076	0.090	0.398	−0.101 – 0.254	1.85		1.85	0.385	0.580
	WM	0.079	0.065	0.224	−0.048 – 0.205	1.00		1.00	2.689	0.101
	IQ	−0.015	0.091	0.865	0.193 – 0.163	NA		NA	NA	NA
Exposed ≥ 14 days (n = 35)	Area	−0.305	0.173	0.079	−0.645 – 0.035	1.44		1.44	1.043	0.231
	Volume	−0.290	0.174	0.097	−0.632 – 0.052	1.00		1.00	4.576	0.033
	Thickness	0.066	0.176	0.710	−0.280 – 0.411	1.00		1.00	3.379	0.067
	WM	0.099	0.131	0.449	−0.157 – 0.356	1.00		1.00	3.837	0.051
	IQ	−0.401	0.175	**0.023**	−0.744 – −0.058	NA		NA	NA	NA
Exposed in all trimesters (n = 21)	Area	−0.447	0.208	**0.032**	−0.854 – −0.040	1.11		1.11	1.993	0.134
	Volume	−0.424	0.209	**0.043**	−0.835 – −0.014	1.00		1.00	1.496	0.222
	Thickness	0.081	0.210	0.700	−0.331 – 0.493	1.00		1.00	0.755	0.385
	WM	0.022	0.156	0.885	−0.282 – 0.493	1.12		1.12	0.341	0.532
	IQ	−0.380	0.209	0.071	−0.790 – 0.030	NA		NA	NA	NA

a Effective degrees of freedom (edf) is an index of the deviation of linearity (linear =1). Bold: pre FDR-corrected significant p-value< .05. Note that no significant differences survived after FDR-correction (applied across 15 tests per effect).

#### 2.7.2 Vertex-wise LMEs

To test the effect of prenatal paracetamol exposure on structural brain development, vertex-wise LMEs were run with FreeSurfer 6.0 (Bernal-Rusiel et al., 2013a, Bernal-Rusiel et al., 2013b), testing group-differences between non-exposed children (n = 254; 512 scans) and children exposed to paracetamol *in utero* (n = 193; 393 scans). We tested group-differences in cortical (1) area, (2) volume and (3) apparent thickness, controlling for the same covariates as before with the addition of child’s baseline age at assessment and time since baseline. The LMEs were repeated for the groups of children exposed to paracetamol for ≥ 14 days (n = 35; 72 scans) and in all three trimesters (n = 21; 44 scans). The vertex-level LMEs were run to identify spatially localized effects of exposure group across the cortex. LMEs take advantage of the longitudinal observations by utilizing the increased statistical power afforded by the repeated measures to detect brain structural differences. Random intercepts account for correlation between repeated brain measures in the same individual. To control for multiple comparisons across space, all surface results were tested against an empirical null distribution of maximum cluster size across 10,000 iterations using Z Monte Carlo simulations (Hagler et al., 2006), using a cluster-forming threshold and cluster-corrected threshold of p < .01.

In addition, vertex-wise LMEs were run to estimate the difference in *change* in brain characteristics (cortical area, volume and thickness), via the group x time interaction (all other covariates comparable). For validation purposes, LMEs were re-run without the birth weight covariate because it is not an antecedent of exposure.

To visualize the exposure group effect across significant clusters, we extracted data from the relevant metric for each child, spatially averaging across vertices in significant clusters. Where multiple clusters were identified, the average across clusters was used to visualize the results. Effect sizes for vertex-wise LME findings were quantified by running comparable LMEs on the extracted clusters, with standardized beta coefficients (Std.Beta) reported as effect sizes in units of standard deviations. Finally, we post-hoc tested for group-differences in the smooth term of age between non-exposed and exposed children (age x exposure) within clusters via comparable GAMMs as before, in order to assess whether exposure-related differences reflected baseline (offset) effects or differences in developmental slopes. FDR-correction was applied across these 7 tests, with significance considered at p(FDR) < .05.

## 3 Results

### 3.1 Age trajectories

**Fig. 2:**
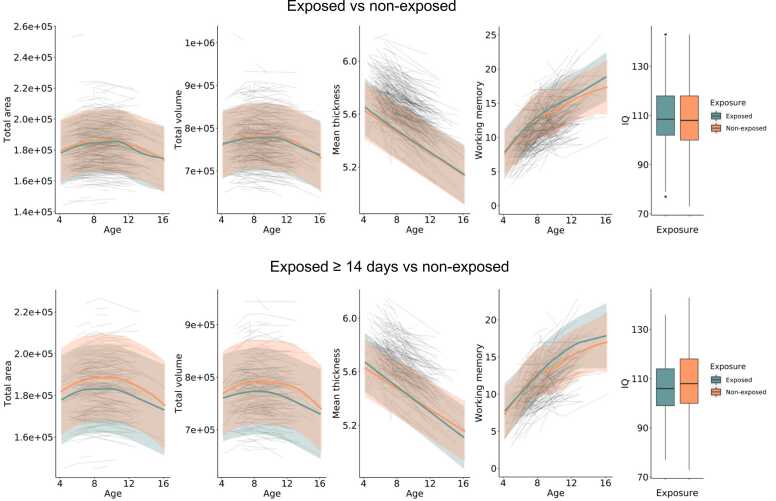
**Age trajectories for structural brain development and cognitive function for exposed and non-exposed children.** The age trajectories for total cortical surface area (mm²), total volume (mm³), mean thickness (mm) and working memory capacity (Digit Span total) was estimated by a smoothing curve over age with a GAMM. Box plots illustrate IQ scores. From the top row: (1) Age trajectories for all the exposed children (n = 193) and non-exposed children (n = 254), and (2) for children exposed for ≥ 14 days (n = 35) and non-exposed children (n = 254). The non-exposed group is in orange and the exposed group is in green.

Fig. 2 shows the age trajectories across global cortical brain measures and WM for non-exposed children (n = 254; 512 scans) with (1) all children exposed to paracetamol *in utero* (n = 193; 393 scans) and (2) children exposed ≥ 14 days *in utero* (n = 35, 72 scans). Results for the group of children exposed in all three trimesters (n = 21, 44 scans) are shown in Supplementary eFigure 1. As expected in the MoBaNeuroCog sample, all total brain measures and WM, were significantly non-linearly related to age, as indicated by estimated degrees of freedom (edf) substantially greater than 1 and highly significant smooth terms (area: edf=5.52, F=143.1, p < .001; volume: edf=7.17, F=78.7, p < .001; thickness: edf=5.23, F=62.0, p < .001; WM: edf =4.74, F=213.4, p < .001). In general, the GAMMs showed no main effect of paracetamol exposure across age, and no significant group-differences in age trajectory slopes, between non-exposed and exposed children (any exposure group), for any of the total brain measures or WM, after FDR-correction (see Table 4). Some pre-corrected significant differences for total surface area and volume were observed in the negative direction between children exposed in all three trimesters (n = 21) and non-exposed. Also, one pre-corrected difference indicative of lower IQ in children exposed ≥ 14 days *in utero* was observed, but this did not survive correction (see Table 4).

### 3.2 Vertex-wise LMEs

**Fig. 3:**
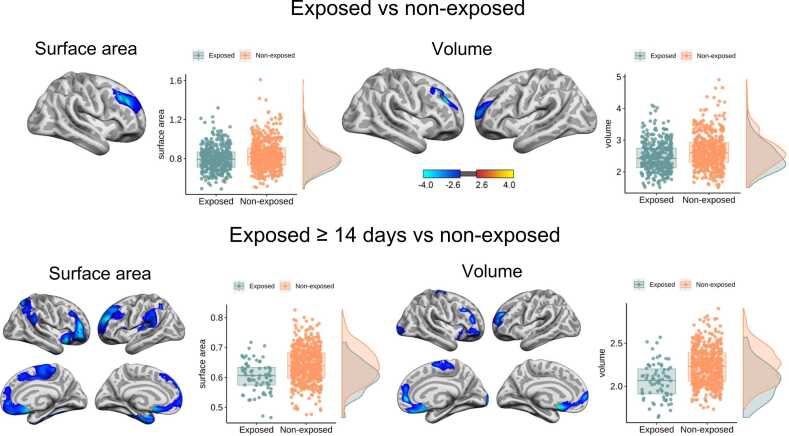
**Relationship between prenatal paracetamol exposed children and non-exposed children on cortical surface area and volume.** From the top row: Significant, cluster-wise corrected (p < .001), clusters from linear mixed models on vertex-wise analyses for (1) all the exposed children (n = 193) and non-exposed children (n = 254), and (2) children exposed for ≥ 14 days (n = 35) and non-exposed children (n = 254). Blue-cyan indicates a negative relationship between surface area and paracetamol exposure, and volume and paracetamol exposure. For visualization purposes, the boxplots show the average effect of exposure group on surface area and volume across significant clusters for right hemisphere, controlling for the child’s baseline age, sex and scanner. The non-exposed group is illustrated in orange, and the exposed groups are illustrated in green.

For children exposed *in utero* (total exposure group; n = 193; 393 scans), vertex-wise LMEs testing group differences across the cortex showed that prenatal paracetamol exposure was related to significantly smaller surface area in a lateral right frontal region and smaller volume in lateral frontal regions in both hemispheres compared to non-exposed (n = 254; 512 scans), at our permutation-based cluster correction threshold (p_perm_<.01; see Fig. 3). No significant clusters were found for apparent cortical thickness.

For the children exposed for ≥ 14 days *in utero* (n = 35; 72 scans), significantly (p_perm_<.01) smaller surface area and volume were found in bilateral and widespread patterns compared to non-exposed children (see Fig. 3). No significant clusters were found for thickness. Further, for children exposed in all three trimesters (n = 21; 44 scans), a largely similar pattern of significantly (p_perm_<.01) smaller surface area and volume was observed bilaterally, and in this group the results showed a small significant cluster suggesting apparently thicker cortex in left lateral parietal cortex compared to non-exposed (see Supplement eFigure 1). Importantly, although we observed average group differences, brain measures showed largely overlapping distributions between groups, as highlighted in Fig. 3 and in eFigure 1.

Additionally, vertex-wise LMEs showed no significant difference in total brain *change* (group x time) between non-exposed and the exposed children, in any exposure group. Results did not change when including or excluding birth weight as a covariate.

Results from comparable LMEs on the extracted cluster values are in eTable 1 in Supplement. Additionally, post-hoc GAMMs suggested no significant group differences in age-related slopes (after FDR-correction; see eTable 1), indicating that exposure-related group differences observed in the LMEs are best explained by offset (baseline) effects already present at the youngest age assessed (age 4), rather than by different rates of developmental *change*.

## 4 Discussion

In this MRI study of prenatal paracetamol exposure, we observed small differences in cortical surface area and volume between exposed and non-exposed children; these differences were most pronounced in long-term exposure groups (≥14 days of exposure *in utero*, and/or exposure in all three trimesters). Our findings suggest exposure-related group differences observed in the LMEs are best explained by offset (baseline) effects already present at the youngest age assessed (age 4), rather than by different rates of developmental *change.* Importantly, although we observed group differences in structural brain development between exposed and non-exposed children, brain measures showed largely overlapping distributions between groups. For cognitive abilities, we found no significant effects of prenatal paracetamol exposure on WM capacity or IQ scores.

### 4.1 Structural brain development

The age trajectories for all three brain measures are in accordance with previous studies showing nonlinear patterns of structural brain development (Bethlehem et al., 2022, Krogsrud et al., 2021, Mills et al., 2016, Tamnes et al., 2017, Walhovd et al., 2016). Cortical surface area and volume showed a slight increase followed by a gradual decrease in adolescence. The increase in cortical volume has been found to be mostly driven by surface area (Amlien et al., 2016). Cortical thickness decreased from 4 to 16 years of age, likely reflecting processes such as synaptic pruning and increasing myelination (Huttenlocher, 1990, Huttenlocher, 1979, Natu et al., 2019). Increased myelin during development has been suggested to change the gray–white matter contrast in MR images resulting in apparent cortical “thinning” (Natu et al., 2019).

### 4.2 Cognitive abilities

WM capacity increased with age, but no effect of prenatal paracetamol exposure on WM capacity was observed. The age-related increase in WM capacity aligns with findings from a previous study, which also included the MoBaNeuroCog sample and showed an association between greater WM function during development and an apparently thinner cortex (Krogsrud et al., 2021).

For IQ, there were no significant group differences between non-exposed and exposed children (any exposure group). The mean IQ score was 2 and 3 points lower for the group of children exposed for ≥ 14 days and in all three trimesters, respectively, compared to non-exposed children. Still, the distributions of scores between groups were largely overlapping, and these results did not survive FDR-correction. A larger difference in IQ scores between exposed and non-exposed has been reported in The Danish National Birth Cohort study where maternal paracetamol use during pregnancy was associated with 3.4 points lower performance IQ in 5-year old children (Liew et al., 2016b). Importantly, IQ scores were not affected if mothers used paracetamol to treat fever, pointing to confounding by indication. Also, children born to mothers reporting fever without using paracetamol scored 4.3 points lower on performance IQ (Liew et al., 2016b).

### 4.3 Paracetamol use for fever

Maternal fever during pregnancy is associated with several adverse child outcomes, such as neural tube defects, brain damage, autism spectrum disorders, lack of task persistence, and poorer academic outcomes (Dombrowski et al., 2003, Edwards, 2006, Gustavson et al., 2019, Hornig et al., 2018). In a study from MoBa, maternal fever was found to be associated with increased risk of offspring ADHD, and the risk of ADHD diagnosis was similar whether the mother had taken paracetamol for their fever or not (Gustavson et al., 2019). Further, short-term use of paracetamol during pregnancy has been associated with reduced risk of offspring ADHD (Gustavson et al., 2021, Ystrom et al., 2017). There has also been reported a reduced risk of autism in the MoBa study when mothers used paracetamol to treat fever during pregnancy (Hornig et al., 2018). In the current study, fever was included as a covariate in all analyses, but we did not have statistical power to investigate effect modification by fever.

### 4.4 Strengths and limitations

To our knowledge, this is the first study that has examined structural brain characteristics after prenatal paracetamol exposure. A strength of the study is the longitudinal measures of brain development and cognitive function, increasing the number of observations in our analyses. Also, the paracetamol exposure information was collected by maternal self-report at several time points during pregnancy, which may have helped minimize exposure misclassification and recall bias compared to a single retrospective assessment. Nonetheless, the absence of direct biological measures of paracetamol exposure, such as levels indexed in meconium (e.g., Baker et al., 2020), is a limitation. Our reliance on maternal self-report means that our exposure estimates may be less precise than those based on meconium or other biomarkers.

The amount and duration of maternal self-reported paracetamol use in the current MoBaNeuroCog subsample and the whole MoBa sample were quite similar. In the MoBa sample, 46 % of pregnant women reported having used paracetamol at least once during pregnancy, and 3.8 % had used for ≥ 28 days (Brandlistuen et al., 2015), while in the MoBaNeuroCog subsample, 43 % of the mothers reported paracetamol use, and 2.9 % had used for ≥ 28 days. A limitation in the current study is the small sample size in the long-term exposure groups. Long-term prenatal paracetamol exposure is most often classified as paracetamol use > 20–28 days (Brandlistuen et al., 2013, Gervin et al., 2017, Gustavson et al., 2021, Ystrom et al., 2017), and several studies report a dose–response association, whereby increased duration of exposure has been associated with increased risk of adverse neurodevelopmental outcomes (Gervin et al., 2017, Liew et al., 2016a, Liew et al., 2014, Liew et al., 2016b, Ystrom et al., 2017). In the current study, only 16 children were exposed for ≥ 21 days, and therefore a≥ 14 day cut-off (n = 35, 18.1 %) was used to reflect longer-term exposure. Similarly, the group exposed in all three trimesters (n = 21, 10.9 %) represented frequent and more continuous exposure throughout pregnancy, which may indicate a cumulative effect of exposure that increases vulnerability to the developing brain (Koehn et al., 2019). However, both the ≥ 14-day and the three-trimester exposure groups were small, and the choice of cut-offs are not meant to reflect clinically meaningful thresholds. Importantly, these analyses were exploratory and not designed to identify “safe” durations or windows of exposure. The small average effects observed in these subgroups must therefore be interpreted with caution, and readers should not draw clinical inferences about specific exposure periods from these results.

In addition, systematic differences have been documented between MoBa participants and the general Norwegian population, with mothers in MoBa being older, more highly educated, and less likely to live alone at the time of delivery (Nilsen et al., 2009). By the 8-year follow-up, the proportion of participants reporting unplanned pregnancies, single parenthood, or smoking during pregnancy had declined relative to baseline (Vejrup et al., 2022). Such selective retention is important to acknowledge, as it constrains the generalizability of our findings to more socioeconomically diverse populations.

### 4.5 Implication of results

The current results indicate that the observed small differences in surface area and volume likely emerged early in life and before 4 years of age. However, whether these differences in brain structure represent a causal effect of prenatal paracetamol exposure, differences in maternal health that increase paracetamol usage, or other unknown factors, cannot be determined by our study. Ystrom et al. (2017) found *paternal* use before pregnancy to represent the same risk for offspring ADHD as maternal use during pregnancy, and Gustavson et al. (2021) found, using a sibling control design, that children of mothers with long-term paracetamol use in *any* pregnancy had increased risk of receiving an ADHD diagnosis; these studies indicate that the association between long-term paracetamol use during pregnancy and ADHD in the child may at least partly be confounded by unmeasured family factors such as genetic transmission (i.e., genetic confounding). Also, long-term use of paracetamol may be more strongly related to unmeasured confounding factors than short-term use.

Future larger, longitudinal studies should include examination of brain structure and function after prenatal and early life paracetamol exposure, while taking into account indications for use and duration of exposure.

## 5 Conclusion

We found a small association between prenatal paracetamol use and structural brain development in children, especially after long-term exposure. However, these associations should be interpreted with caution, as our study cannot distinguish whether the observed differences reflect prenatal paracetamol exposure, underlying maternal health, unmeasured confounding, or chance. No association with IQ and working memory capacity was detected. As with any medication in pregnancy, paracetamol should only be used when clearly needed, and then at the lowest effective dose for the shortest possible duration (European Medicines Agency, 2025).

## CRediT authorship contribution statement

**Eivind Ystrøm:** Writing – review & editing, Supervision, Funding acquisition, Data curation, Conceptualization. **Hedvig Nordeng:** Writing – review & editing, Supervision, Project administration, Funding acquisition, Data curation, Conceptualization. **James Roe:** Writing – review & editing, Visualization, Investigation, Formal analysis. **Janne von Koss Torkildsen:** Writing – review & editing. **Wood Mollie:** Writing – review & editing, Formal analysis. **Stine Kleppe Krogsrud:** Writing – original draft, Visualization, Supervision, Project administration, Investigation, Formal analysis, Data curation, Conceptualization.

## Funding

This work was supported by the European Research Council Starting Grant “DrugsInPregnancy” (grant number 678033). The MoBa Neurocognitive Development project was funded by the Norwegian Research Council. EY was supported by the Research Council of Norway (grant numbers 336078, 288083, and 331640) and the 10.13039/501100000781European Research Council (101045526 and 101073237). JKT was supported by the Research Council of Norway (Grant ID 331640). The Norwegian Mother, Father and Child Cohort Study is supported by the Norwegian Ministry of Health and Care Services and the Ministry of Education and Research.

## Declaration of Competing Interest

The authors declare that they have no known competing financial interests or personal relationships that could have appeared to influence the work reported in this paper.

## Data Availability

The data that has been used is confidential.
