# Supplementary material for: Associations between prenatal paracetamol exposure and brain development from ages 4–16: A longitudinal MRI study
Source: Dev Cogn Neurosci. 2025 Dec 30;78:101667. doi: 10.1016/j.dcn.2025.101667 (PMC12809025; doi:10.1016/j.dcn.2025.101667)
Supplement: Supplementary file 1 — Supplementary material [file mmc1.docx]

**Supplementary Material**

**Associations between prenatal paracetamol exposure and brain development from ages 4 to 16: A longitudinal MRI stud**

Running title: Prenatal paracetamol exposure and brain development

Dr. Stine Kleppe Krogsrud, Prof. Hedvig Nordeng, Dr. James M. Roe, Prof. Janne von Koss Torkildsen, Dr. Mollie Wood, Prof. Eivind Ystrom

**eTable 1.** The effect of paracetamol exposure *in utero* vs non-exposure on significant cluster from vertex-wise LME for surface area, volume and thickness.

**eFigure1.** Relationship between children exposed in all three trimesters *in utero* (n=21) and non-exposed children (n=254) on structural brain development and cognitive function.

**eTable 1. The effect of paracetamol exposure *in utero* vs non-exposure on significant cluster from vertex-wise LME for surface area, volume and thickness.**

|  |  | **The effect of paracetamol exposure *in utero* on clusters across age** | | | | | **Smooth age x exposure** | | | |
| --- | --- | --- | --- | --- | --- | --- | --- | --- | --- | --- |
| Exposure groups | Brain measures | | Std Beta | Std Error | P-value | 95% CI | ^a^Edf | Ref.df | F | P-value |
| Total exposure (n=193) | Area | | -0.257 | 0.089 | 0.004 | -0.431 – -0.082 | 1.00 | 1.00 | 0.747 | 0.388 |
|  | Volume | | -0.327 | 0.092 | < 0.001 | -0.508 – -0.147 | 1.00 | 1.00 | 4.776 | **0.029** |
| Exposed ≥14 days (n=35) | Area | | -0.669 | 0.165 | < 0.001 | -0.994 – -0.345 | 1.00 | 1.00 | 1.401 | 0.237 |
|  | Volume | | -0.813 | 0.172 | < 0.001 | -1.150 – -0.476 | 1.00 | 1.00 | 1.515 | 0.219 |
| Exposed in all trimesters (n=21) | Area | | -0.840 | 0.203 | < 0.001 | -1.237 – -0.443 | 1.00 | 1.00 | 1.937 | 0.165 |
|  | Volume | | -1.121 | 0.216 | < 0.001 | -1.544 – -0.698 | 1.00 | 1.00 | 0.110 | 0.740 |
|  | Thickness | | 0.761 | 0.219 | < 0.001 | 0.332 – 1.190 | 1.00 | 1.00 | 0.828 | 0.363 |

^a^Effective degrees of freedom (edf) is an index of the deviation of linearity (linear = 1).

Bold: pre FDR-corrected significant p-value<.05.

**
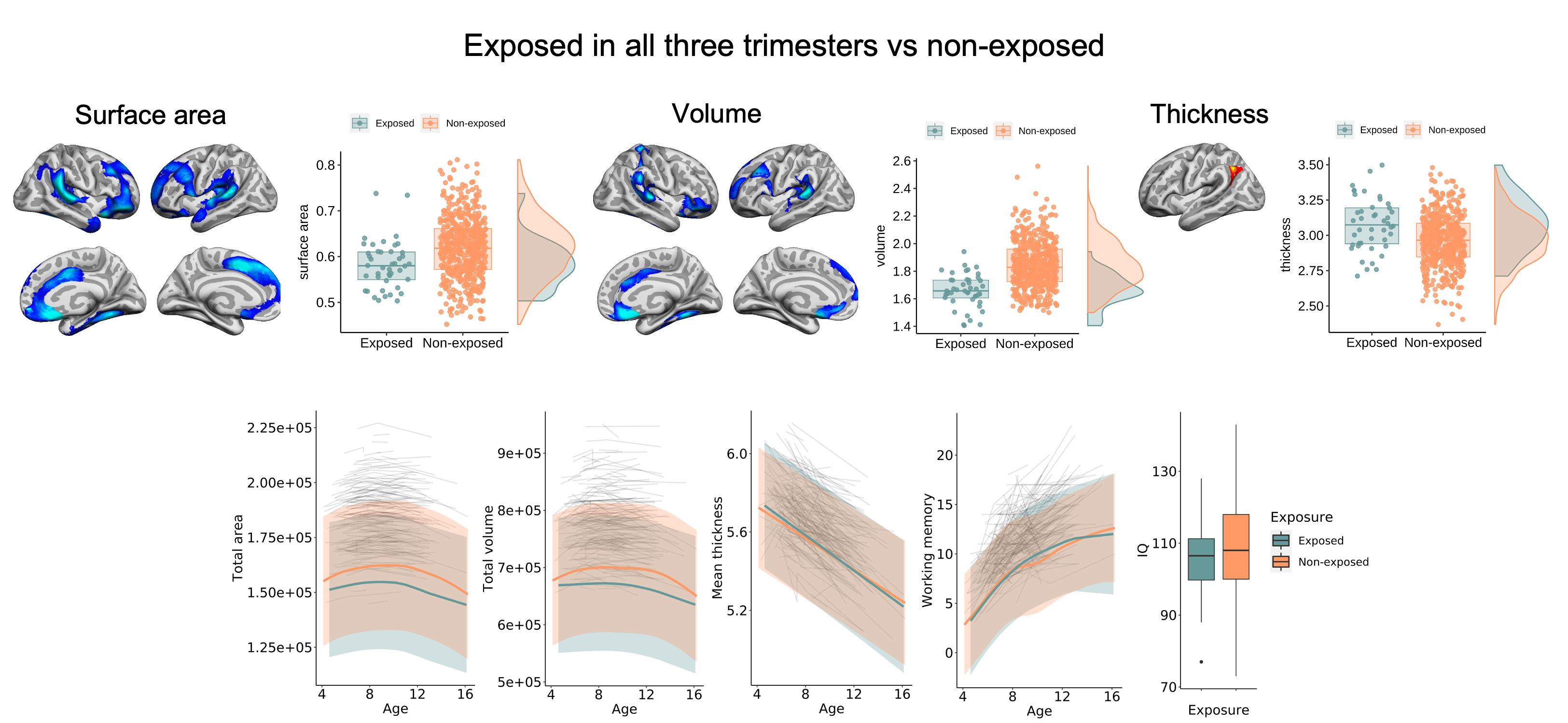
**

**eFigure 1. Relationship between children exposed in all three trimesters *in utero* (n=21) and non-exposed children (n=254) on structural brain development and cognitive function.**
Top row: Significant, cluster-wise corrected (P<.001), clusters from linear mixed models on vertex-wise analyses. Blue-cyan indicates a negative relationship between surface area and paracetamol exposure, and volume and paracetamol exposure. Red-yellow indicates a positive relationship between apparent cortical thickness and prenatal paracetamol exposure. The boxplots visualize show the average effect across significant clusters for cortical surface area, volume and thickness, controlling for the child’s age, sex and scanner. These are based on average effects for the right hemisphere for cortical surface area and volume, and the left hemisphere for thickness.
Bottom row: The age trajectories for total cortical surface area, total volume, mean thickness and working memory performance (Digit Span total) was estimated by a smoothing curve over age with a GAMM. Box plots illustrate IQ scores. The non-exposed group is in orange and the exposed group is in green.
